# Study on the heavy metal bioconcentrations of the Shadegan international wetland mosquitofish, *Gambusia affinis*, by inductively coupled plasma technique

**DOI:** 10.1186/2052-336X-11-22

**Published:** 2013-07-25

**Authors:** Hassan Nasirian, Amir Hossein Mahvi, Mostafa Hosseini, Babak Vazirianzadeh, Sayyed Mohammad Taghi Sadeghi, Shahrokh Nazmara

**Affiliations:** 1 Department of Medical Entomology and Vector Control, School of Public Health, Tehran University of Medical Sciences, Tehran, Iran; 2 Department of Environmental Health Engineering, School of Public Health and Center for Water Quality Research, Institute for Environmental Research, Tehran University of Medical Sciences, Tehran, Iran; 3 Department of Epidemiology and Biostatistics, School of Public Health, Tehran University of Medical Sciences, Tehran, Iran; 4 Department of Medical Entomology and Infectious and Tropical Diseases Research Center, School of Public Health, Ahvaz Jundishapur University of Medical Sciences, Ahvaz, Iran; 5 Department of Environmental Health Engineering, School of Public Health, Tehran University of Medical Sciences, Tehran, Iran

**Keywords:** *Gambusia affinis*, Bioconcentration, Heavy metal, Inductively coupled plasma, Mosquitofish, Wetland

## Abstract

The purpose of this study was to evaluate the levels of heavy metal bioconcentration of the mosquitofish (*Gambusia affinis*) in Shadegan international wetland. Sampling including the water, waterbed sediment and mosquitofish was carried out from the selected sampling sites during October and November 2011, and analyzed by the ICP-OES. Results show that the water has poor qualitative condition, according to EPA and WHO water quality standards. The level of the water Cr in the selected sites in both months and the levels of Fe, Mn and Zn during October in the SW_1_ site were higher than the instrumental detection limits indicating that the water was contaminated with these metals in the mentioned sites and months. The levels of the waterbed sediment As, Co, Cr, Cu, Fe, Mn, Pb and Zn, and mosquitofish Cr, Cu, Fe, Mn, Zn, Co and Cd were much higher than the instrumental detection limits, indicating that the waterbed sediment and mosquitofish were contaminated with them during October and November in the selected sites. Statistical assessments reveal that there is a significant difference between the mentioned contaminated water, waterbed sediment and mosquitofish heavy metals (all *P*-values < 0.05). In overall, it is considered that the contaminated heavy metals can be accumulated in the waterbed sediment and bioconcentrated in the wildlife tissues, then finally can be entered in the marine food chains and biomagnified there after long periods. In conclusion, this paper confirmed that the *G. affinis* can be used as a bioindicator of heavy metal pollution in marine ecosystems such as wetlands.

## Introduction

Mosquitofish, *Gambusia affinis* (Actinopterygii: Cyprinodontiformes: Poeciliidae) (Baird and Girardi, 1853), is a top-feeding minnow and is a known good larvivorous fish with wide distribution in countries in the Eastern Mediterranean Region. It is unique in its global distribution and is small, tiny, grey or greyish black, measuring up to 4–6 cm in length. The fish is viviparous (lays young ones and not eggs), breeds prolifically and requires no special egg-laying site [1], with an extraordinary ability to tolerate and use a wide range of natural and artificial conditions [2,3]. Recognizing the high larvivorous potential of *G. affinis*, this fish species was purposely introduced from its native Texas (southern USA) to the Hawaiian Islands in 1905 and Spain in 1921, then from there into Italy during the 1920s and later to 60 other countries [4]. It is reputed to have shown potential to reduce mosquito populations throughout the world [1] and has been used in many parts of the world to control mosquito larvae. In Iran, *G. affinis* were introduced from Italy into the Ghazian marshes, Caspian littoral during 1922–1930 and after initial technical problems, were successfully used in combating malaria [5]. Beginning in 1966, intensive efforts were made to introduce the fish in the whole country [6]. Recently Gouya reported that *Gambusia* has been used for the last 40 years in Sistan and Baluchistan province [1].

Chemical analysis of the environment matrix such as water or sediment is the most direct approach to reveal the heavy metal pollution status in the environment, while it cannot afford the powerful evidence on the integrated influence and possible toxicity of such pollution on the organisms and ecosystem. Biomonitoring is a scientific technique for assessing environment pollutions. Fishes and seabirds are often monitored for the presence of environment contaminants [7] such as heavy metals. Heavy metals are one of the major and most widespread groups of contaminants because of their persistent nature and slow elimination from environmental compartments [8-10]. Fish has attracted much attention in the biomonitoring of water pollution due to its special biological characters such as relatively long life cycle, easy to raise etc. More importantly, fish species are at the top position in the aquatic food chains and may directly affect the health of humans, which makes it much of significance for the biomonitoring using fish [7]. In this regard, the *G. affinis* can be used as a multi-purpose, such as malaria combating and biomonitoring. Some studies such as the blood of the male *G. affinis* has been served as a useful biomarker for assessing previous exposure to estrogenic compounds [11], oxidative stress and locomotor behaviour response of *G. affinis* has been used as biomarkers in the pesticide contaminated aquatic streams [12] and western mosquitofish has been used as a bioindicator of exposure to organochlorine compounds [13] confirmed that *G. affinis* can be used as a biomarkers or bioindicators.

More and more attention has been drawn due to the wide occurrence of heavy metal pollution in the aquatic system [8-10]. Interestingly, small amounts of these elements are common in our environment and diet and are actually necessary for good health, but large amounts of any of them may cause acute or chronic toxicity (poisoning). It is obvious that toxic metals are elements and they cannot be destroyed and may be made insoluble in the body and in the food chains. Some heavy metals may transform into the persistent metallic compounds with high toxicity, which can be bioaccumulated in the organisms, magnified in the food chains, thus threatening human health. Living organisms require varying amounts of heavy metals but excessive levels can be damaging to the organism and may cause vital effect. Some studies conducted about heavy metals on *G. affinis*. In a study was proved that Cd body content of the *G. affinis* was increased much higher from water than the food [14,15]. Klerks and Lentz (1998) reported that the tissue metal levels of mosquitofish were highly elevated for lead and (to a lesser extent) for zinc in a contaminated habitat [16]. Franssen (2009) studied the effects of heavy metal mine drainage on population size structure, reproduction, and condition of *G. affinis*[17]. In Iran, Taghavi Jelodar and Hosseinzadeh Colagar (2011) in a study confirmed that the average Cr, Ni, Cd and Pb concentrations were found higher in female than the male *G. affinis* samples [18]. The induction of micronuclei and nuclear abnormalities in erythrocytes and Cu and Cd accumulation in whole body of *G. affinis* were studied by Güner *et al*. (2011) [19].

Wetlands take on characteristics that distinguish it as a distinct [20] and the most productive among the world ecosystems [21]. Shadegan international wetland in Khuzestan province, south western of Iran, is considered to be one of the most wonderful natural attractions of the world because of its unique biodiversity and it’s linking to Jarahi river and Persian Gulf waters. The high diversity of plant and animal species in Shadegan wetland has caused the International Supreme Council for the Environment to register it as an international protected zone. In Shadegan wetland there are different fish varieties which live in its water such as *G. affinis*. Although Shadegan international wetland has the potential to become a tourism destination and bears many socioeconomic advantages for local residents, different kinds of pollutants such as heavy metals are threatening its ecosystem. It is facing a series of environmental crises largely caused by an oil spill, inflow of fertilizers and the release of sewage produced by nearby factories. The discharge of more than 50 million cubic meters of industrial and urban wastes which likely contain heavy metals are released into it are threatened its wildlife and nature ecosystem. Thus measurement of the heavy metals which exist in the waterbed sediment, water and animal tissues such as *G. affinis* that lives in its water can be taken by regular environmental surveys to reduce heavy metal pollutions. The objective of this survey was to evaluate the quantity of some heavy metals on Shadegan wetland mosquitofish.

## Materials and methods

The experiment described in this work was approved by Tehran University of Medical Sciences and conducted on the whole dead fish bodies which were caught by a special net as fish caught for human food so there is no any stress to the animals.

### Geographical information

This study was conducted in Shadegan international wetland area in Khuzestan province, south western of Iran which is one of the 18 international wetlands registered on UNESCO’s Natural Heritage List. It is the largest wetland in Iran, which covers an area of 400,000 hectares, located 52 km far from Abadan and 40 km far from Ahvaz (the capital city of Khuzestan province) and is surrounded from north to Shadegan city and Khor Doraq, from south to Bahmanshir river, from west to Darkhovien and Abadan road and from east to Khure-Musa, its surface is covered by great varieties of vegetations. Its water supply is mainly through Karoun river. The area has a hot and humid climate and its coordinates are: 48° 17^’^- 48° 50^’^E 30° 17^’^- 30° 58^’^N.

### Site selection

Samples were collected from two different selected sites of the Shadegan international wetland in the freshwater part which their water depths were low and mosquitofish were found with very high density. The first, SW_1_, site located at the east of the wetland between Shadegan city and wetland where urban waste released into the wetland. The second, SW_2_, site is selected from a neighboring villages of the wetland, Ragbeh and Sarakhieh, located at the 10 Km of the Shadegan-Darkhovein road.

### Samplings

The parameters which measured during sampling were water temperature, electrical conductivity (EC), dissolved oxygen (DO), pH, hardness, total dissolved solids (TDS), total suspended solids (TSS), turbidity, and salinity using the HQ40d Portable Multi-Parameter Meter, titration method and turbidity meter.

Since the precipitation and runoff waters have been finished in the autumn and also the rainfall of the next growing season has not been started yet, as well as the water evaporates has reached at its maximum in the end of the summer. So it seems that heavy metal pollutions are at their peak which the sampling was attempted. Sampling of water, waterbed sediment, and mosquitofish was carried out from the same selected sampling sites, SW_1_ and SW_2_, during late October to late November 2011. One sample of the waterbed sediment, water and mosquitofish per month from each sampling sites were collected. Acid-washed watchglass were used for the grab collection and the samples were stored at 0°C in acid-washed (10% nitric acid) polypropylene tubes. Mosquitofish collecting was done by a modified student insect net during the period of a peak activity of mosquitofish in the selected sites. The samples were collected with the minimum amount of water using sterilized polypropylene tubes. A sufficient amount of 95% ethanol was add and preserved at 0°C before taking to the laboratory.

### Heavy metal isolation and analysis procedure

#### Mosquitofish preparation samples

A revised procedure by Lynch et al. (1988) was followed for mosquitofish sample preparation and chemical analysis [22]. Because of mosquitofish has a small body size, as mentioned in the introduction, the all their both males and females bodies were used for sample preparation. The samples were oven-dried for 24 hrs, then keeping in desiccator for 24 hours, the mosquitofish were acid digested with redistilled nitric acid and hydrogen peroxide (H2O2). The dried samples were placed in 50 ml glass beakers with 10 ml of concentrated nitric acid (HNO3) for concentrations of 1.0 g. Each mixture was gently heated for 1 hr, allowed to cool, then 5 ml of 30% H2O2 was added and heated gradually to boil (approximately 10 min) and 5 ml of nitric acid was added. The solution was concentrated to 10 ml by heating. The cooled resulting solutions were passed through a 0.2 micrometer (μm) membrane filter into polyethylene bottles and diluted with double de-ionized water (DDI water) to various volumes within the linear range of the inductively coupled plasma (ICP) for analysis. The entire digestion process was done in a fume hood. Samples were stored at room temperature (about 25°C) until analysis. All glasswares and equipments were pre-cleaned with 10% nitric acid, then rinsed with high-purity DDI water before and after each digestion process to avoid cross contamination of samples and biasing of the results.

#### Waterbed sediment preparation samples

The waterbed sediment samples were oven-dried to a constant weight at 60°C for 24 hrs in order to prevent the loss of possible volatile metallic compounds, and to facilitate sample grinding and sieving. The samples were later homogenized by grinding in a mortar and pestle. The mortar, pestle, and sieve were cleaned before and after every sample with 10% HNO3 and rinsed with high-purity DDI water. Digestion and analytes extraction for ICP-MS analysis were performed using an acid mixture procedure [23]. One gram of each sediment sample was precisely measured and transferred into a 50 ml glass beaker, then 4 ml of HNO3 (1 + 1) and 10 ml of HCl (1 + 4) were added and the solution was covered with a watch glass. The beaker was then placed on a hotplate for extraction of the analytes at an adjusted reflux temperature of 95°C. The sample was heated for two hours while avoiding vigorous boiling of the solution (though very slight boiling could be tolerated) under a fume hood. The solution was then reduced to 10 ml by boiling, followed by cooling. The cooled solutions were passed through a 0.2 micrometer (μm) membrane filter into polyethylene bottles and diluted with DDI water to various volumes within the linear range of the inductively coupled plasma (ICP) for analysis. Samples were analyzed as soon as possible to minimize the effect of the various matrices on the stability of the diluted samples.

#### ICP-OES analysis

The prepared laboratory samples for metals testing including As, Cd, Co, Cr, Cu, Fe, Hg, Mn, Pb and Zn were subjected to ICP-OES (Germany SPECTRO Company, Spectro atcos Model) instrument to quantify the composition of the given samples.

#### Statistical analysis

Data from the investigated heavy metal concentration between the different selected sites and dates, and between the water, waterbed sediment and mosquitofish samples were analyzed by Mann–Whitney U-test and Wilcoxon Signed Ranks test, and Mann–Whitney U-test and Kruskal-Wallis test, respectively using PASW Statistics 18 version software. One-Sample T Test was used for comparing the water quality parameters with Environmental Protection Agency (EPA) and WHO standards, and water, waterbed sediment and mosquitofish heavy metal contaminated with EPA and WHO water and soil standards [24-26].

## Results

This study was conducted in the two selected sites, SW_1_ and SW_2_, of the Shadegan international wetland area in Khuzestan province, south western of Iran, during the late October to late November 2011. The water quality parameters which measured during sampling were water temperature, electrical conductivity (EC), dissolved oxygen (DO), pH, hardness, total dissolved solids (TDS), total suspended solids (TSS), turbidity, and salinity. The prepared laboratory of the water, waterbed sediment, and mosquitofish samples for heavy metal concentrations including As, Cd, Co, Cr, Cu, Fe, Hg, Mn, Pb and Zn were subjected to ICP-OES instrument to quantify the composition of the given heavy metal concentrations samples.

### Water parameters determination

**Figure 1:**
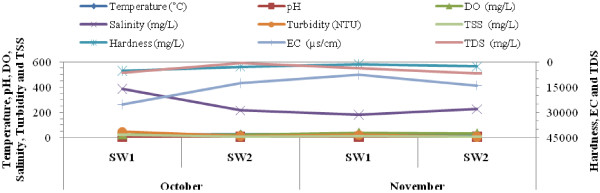
Water parameters measured during sampling in the selected sites of the Shadegan international wetland from the late October to late November 2011.

Table 1 and Figure 1 show the parameters which measured from the selected sites, SW_1_ and SW_2_, during sampling from the late October to late November 2011. In overall, as shown in the Table 1 and Figure 1, the parameters of the water quality have poor condition, according to EPA and WHO water quality standards which led to increasing pollution in the Shadegan international wetland. The electrical conductivity (EC), hardness, total dissolved solids (TDS), total suspended solids (TSS) and turbidity were higher ranges than the EPA and WHO water quality standards [26,27]. One-Sample T Test indicated that there was a significant difference between these water quality parameters and the EPA and WHO standards (all *P*-values < 0.05) [26,27].

**Table 1:** Water parameters measured during sampling in the selected sites of the Shadegan international wetland from the late October to late November 2011

**Month**	**Site**	**Temperature (°C)**	**pH**	**DO**	**Salinity**	**Hardness**	**TDS**	**TSS**	**Turbidity (NTU)**	**EC (μs/cm)**
						**(mg/L)**				
October	SW_1_	23.6	7.4	25	386.7	5225	6290	21	47	25260
	SW_2_	26.8	7.6	25	215.38	2826.2	676	6	14	12650
November	SW_1_	21.5	7.8	41	181	1343.5	3860	14.7	21	7740
	SW_2_	24.2	7.6	35	226	2787.5	6980	8.2	8	14000
Mean (Std. deviation)		24.0 (2.2)	7.6 (0.16)	31.5 (7.9)	252.3 (91.7)	3045.6 (1608.5)	4451.5 (2850.6)	12.5 (6.8)	22.5 (17.2)	14912.5 (7404.2)

### Instrumental detection limits

**Figure 2:**
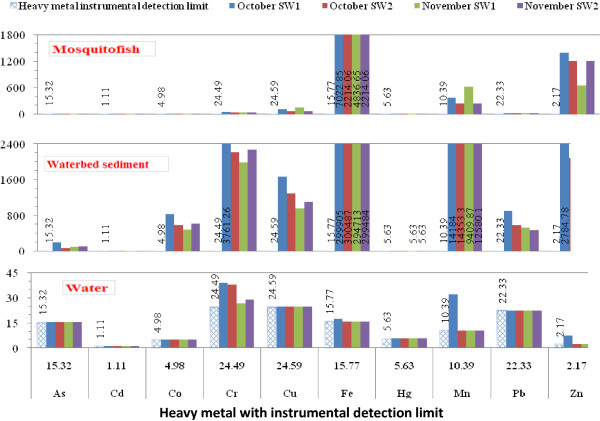
Heavy metals investigated in the water, waterbed sediment and mosquitofish samples (μg/L) in the selected sites of the Shadegan international wetland from the late October to late November 2011.

**Figure 3:**
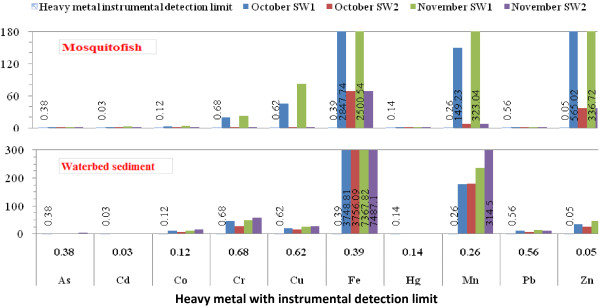
**Heavy metals investigated in the waterbed sediment and mosquitofish samples (μg/g) in the selected sites of the Shadegan international wetland from the late October to late November 2011.**

The instrumental detection limits of investigated heavy metals were shown in Tables 2 &3 and Figures 2 &3 by μg/L and μg/g according to the water, waterbed sediment, and mosquitofish sample preparation and analysis in the materials and methods.

**Table 2:** Heavy metals investigated in the water, waterbed sediment and mosquitofish (μg/L) in the selected sites of the Shadegan international wetland from the late October to late November 2011

**Mater**	**Month**	**Site**	**Heavy metal**									
			**As**	**Cd**	**Co**	**Cr**	**Cu**	**Fe**	**Hg**	**Mn**	**Pb**	**Zn**
water	October	SW_1_	<15.32	<1.11	<4.98	**39.15**	<24.59	**17.44**	<0.36	**32.13**	<22.33	**7.40**
		SW_2_	<15.32	<1.11	<4.98	**38.06**	<24.59	<15.77	<0.35	<10.39	<22.33	<2.17
	November	SW_1_	<15.32	<1.11	<4.98	**26.74**	<24.59	<15.77	<5.63	<10.39	<22.33	<2.17
		SW_2_	<15.32	<1.11	<4.98	**29.18**	<24.59	<15.77	<5.63	<10.39	<22.33	<2.17
Mean (Std. deviation)			-	-	-	**33.3 (6.2)**	-	-	-	-	-	-
Waterbed sediment	October	SW_1_	**195. 9**	<1.11	**831.6**	**3761.3**	**1665.1**	**299905**	<5.63	**14184.0**	**897.3**	**2784. 8**
		SW_2_	**68.8**	<1.11	**582.9**	**2212.8**	**1289.4**	**300487**	<5.63	**14353.3**	**579.5**	**2085.2**
	November	SW_1_	**97.5**	<1.11	**483.6**	**1985. 9**	**964.01**	**294713**	<5.63	**9409.9**	**529.6**	**1887.9**
		SW_2_	**111.9**	<1.11	**617.1**	**2268.5**	**1106.6**	**299484**	<5.63	**12580.1**	**469. 7**	**1549.8**
Mean (Std. deviation)			**118.5 (54.6)**	**-**	**628.8 (146.6)**	**2557.1 (812.0)**	**1256.3 (303.4)**	**298647.2 (2654.9)**	**-**	**12631.8 (2291.7)**	**616.0 (190.9)**	**2076.9 (521.1)**
Mosquitofish	October	SW_1_	<15.32	<1.11	**8.01**	**50.14**	**111.49**	**7022.85**	<5.63	**368.02**	<22.33	**1393.4**
		SW_2_	<15.32	<1.11	<4.98	**33.59**	**63.94**	**2214.06**	<5.63	**237.31**	26.42	**1199.1**
	November	SW_1_	<15.32	**5.19**	**8.69**	**43.33**	**159.71**	**4836.65**	<5.63	**624.83**	<22.33	**651.3**
		SW_2_	<15.32	<1.11	<4.98	**33.59**	**63.94**	**2214.06**	<5.63	**237.31**	26.42	**1199.1**
Mean (Std. deviation)			-	-	-	**40.2 (8.1)**	**99.8 (45.8)**	**4071.9 (2323.5)**	-	**366.9 (182.7)**	-	**1110.7 (319.7)**
Instrumental detection limit			15.32	1.11	4.98	24.49	24.59	15.77	5.63	10.39	22.33	2.17

**Table 3:** **Heavy metals investigated in the waterbed sediment and mosquitofish (**μ**g/g) in the selected sites of the Shadegan international wetland from the late October to late November 2011**

**Mater**	**Month**	**Site**	**Heavy metal**									
			**As**	**Cd**	**Co**	**Cr**	**Cu**	**Fe**	**Hg**	**Mn**	**Pb**	**Zn**
Waterbed sediment	October	SW_1_	**2.45**	<0.03	**10.40**	**47.02**	**20.81**	**3748.81**	<0.14	**177.30**	**11.22**	**34.81**
		SW_2_	**0.86**	<0.03	**7.29**	**27.66**	**16.12**	**3756.09**	<0.14	**179.42**	**7.24**	**26.06**
	November	SW_1_	**2.44**	<0.03	**12.09**	**49.65**	**24.10**	**7367.82**	<0.14	**235.25**	**13.24**	**47.20**
		SW_2_	**2.81**	<0.03	**15.43**	**56.72**	**27.66**	**7487.10**	<0.14	**314.50**	**11.74**	**38.75**
Mean (Std. deviation)			**2.1 (0.87)**	-	**11.3 (3.4)**	**45.3 (12.4)**	**22.2 (4.9)**	**5589.96 (2122.3)**	-	**226.6 (64.4)**	**10.9 (2.6)**	**36.7 (8.9)**
Mosquitofish	October	SW_1_	<0.38	<0.03	**3.25**	**20.33**	**45.21**	**2847.74**	<0.14	**149.23**	<0.56	**565.02**
		SW_2_	<0.38	<0.03	<0.12	**1.05**	**2.00**	**69.28**	<0.14	**7.42**	0.83	**37.52**
	November	SW_1_	<0.38	**2.68**	**4.50**	**22.40**	**82.57**	**2500.54**	<0.14	**323.04**	<0.56	**336.72**
		SW_2_	<0.38	<0.03	<0.12	**1.05**	**2.00**	**69.28**	<0.14	**7.42**	0.83	**37.52**
Mean (Std. deviation)			-	-	-	**11.2 (11.8)**	**32.9 (38.8)**	**1371.7 (1510.6)**	-	**121.8 (149.9)**	-	**244.2 (256.2)**
Instrumental detection limit			0.38	0.03	0.12	0.68	0.62	0.39	0.14	0.26	0.56	0.05

### Water, waterbed sediment and mosquitofish heavy metal isolation and analysis

Tables 2 &3 and Figures 2 and 3 show the water, waterbed sediment and mosquitofish investigated heavy metals by μg/L and μg/g in selected sites, SW_1_ and SW_2_, during the late October to late November 2011. The levels of the water As, Cd, Co, Cu, Hg and Pb, waterbed sediment Cd and Hg, and mosquitofish As, Hg and Pb were less than the instrumental detection limits (Tables 2 &3 and Figures 2 &3).

### Water heavy metal isolation and analysis

As shown in the Table 2 and Figure 2, the level of the water Cr is slightly above the instrumental detection limits in the selected sites during October and November (Table 2 & Figure 2) and the level of the Fe is the same as Cr but only in the SW_1_ site during October (shown as bold font style in Table 2). The levels of the water Mn and Zn are three times higher than the instrumental detection limits in the SW_1_ site during October (shown as bold font style in Table 2). In other words, it means that the water was polluted with Cr, Fe, Mn and Zn investigated heavy metal in this study in the mentioned sites and months.

### Waterbed sediment heavy metal isolation and analysis

According to heavy metal instrumental detection limits (Tables 2 &3 and Figures 2 &3) the levels of all heavy metals except Cd and Hg are much higher than the heavy metal instrumental detection limits by μg/L and μg/g in waterbed sediment during October and November in the selected sites (shown as bold font style in Tables 2 &3), it means that the waterbed sediment was polluted with all heavy metal investigated except Cd and Hg in this study during October and November in the selected sites.

### Mosquitofish heavy metal isolation and analysis

The levels of the mosquitofish Cr, Cu, Fe, Mn and Zn are much higher than the heavy metal instrumental detection limits by μg/L and μg/g during October and November in the selected sites (shown as bold font style in Tables 2 &3). Also the level of the mosquitofish Co is higher than the heavy metal instrumental detection limits by μg/L and μg/g during October and November only in the SW_1_ site (shown as bold font style in Tables 2 &3). The *G. affinis* Cd quantity is much higher than the heavy metal instrumental detection limits only during November in the SW_1_ site (shown as bold font style in Tables 2 &3). In other words, it means that the mosquitofish was polluted with Cr, Cu, Fe, Mn, Zn, Co and Cd investigated heavy metal in this study in the mentioned sites and months.

### Water, waterbed sediment and mosquitofish heavy metal statistical assessments

Statistical assessments for all heavy metal concentrations reveal that there is no significant differences between the two sampling dates at the selected sites, using the Wilcoxon Signed Ranks test and the Mann–Whitney U-test, respectively (all *P*-values > 0.05) (Table 4). Kruskal-Wallis test indicates that there is a significant differences between the water, waterbed sediment and mosquitofish heavy metal concentrations for all heavy metals (shown as bold font style in Table 4) except Cd and Hg by μg/L (all *P*-values < 0.05 except for Cd and Hg *P*-values > 0.05) (Table 4). Also Mann–Whitney U-test shows that there is a significant difference between the waterbed sediment and mosquitofish As, Co, Cr, Fe and Pb heavy metal concentrations by μg/g (all *P*-values < 0.05) (shown as bold font style in Table 4), whereas there is no significant difference between the waterbed sediment and mosquitofish Cd, Cu, Hg, Mn and Zn heavy metal concentrations by μg/g (all *P*-values > 0.05) (Table 4).

**Table 4:** **Significance level ( *P *-values) of statistical analysis comparing investigated heavy metal concentration between the site and date sampling, and between the water, waterbed sediment and mosquitofish samples of the Shadegan international wetland**

	**Heavy metal**									
	**As**	**Cd**	**Co**	**Cr**	**Cu**	**Fe**	**Hg**	**Mn**	**Pb**	**Zn**
Between sites (SW1 and SW2) (Mann–Whitney U-test)	0.94	0.35	0.54	0.51	0.36	0.50	0.97	0.40	0.76	0.61
Between months(October and November) (Wilcoxon Signed Ranks test)	1.0	0.18	0.34	0.78	0.92	0.61	0.18	1.0	0.46	0.13
Between water, waterbed sediment and mosquitofish (Kruskal-Wallis test)	**0.005**	0.37	**0.01**	**0.02**	**0.006**	**0.007**	0.11	**0.007**	**0.01**	**0.007**
Between waterbed sediment and mosquitofish (Mann–Whitney U-test)	**0.01**	0.32	**0.02**	**0.02**	1.0	**0.02**	1.0	0.24	**0.02**	0.24

One-Sample T Test indicates that there is a significant difference between the water Cr, Fe, Mn and Zn, waterbed sediment As, Co, Cr, Cu, Fe, Mn, Pb and Zn, and mosquitofish Cr, Cu, Fe, Mn, Zn, Co and Cd heavy metal investigated pollutions and the EPA and WHO water and soil standards (all *P*-values < 0.05) [24-26].

## Discussion

In overall, as shown in the Table 1 and Figure 1, the parameters of the water quality have poor condition, according to EPA and WHO water quality standards which led to increasing pollution in the Shadegan international wetland and confirmed by One-Sample T Test that showed a significant difference between these water quality parameters and the EPA and WHO standards (all *P*-values < 0.05) [26,27].

Although, the level of the water Cr in both months in the two selected sites and the levels of water Fe, Mn and Zn during October in SW_1_ site are higher than the instrumental detection limits by μg/L (Table 2 & Figure 2), indicated that the water was polluted with Cr, Fe, Mn and Zn investigated heavy metal in the mentioned sites and months in this study, it can be considered that to be accumulated in the waterbed sediment and bioconcentrated in the wildlife and animal tissues such as *G. affinis* that lives in the wetland water as shown in the Tables 2 &3 and Figures 2 and 3 and led to increase heavy metal pollution because, finally they entered in the marine food chains, and biomagnified there after long periods.

According to the heavy metal instrumental detection limits (Tables 2 &3 and Figures 2 &3) the levels of all heavy metals except Cd and Hg are much higher than the instrumental detection limits by μg/L and μg/g for the waterbed sediment during October and November in the SW_1_ and SW_2_ sites (shown as bold font style in Tables 2 &3) indicated that the waterbed sediment was polluted with all heavy metal investigated except Cd and Hg during October and November in selected sites in this study.

As indicated in the Tables 2 &3 and Figures 2 and 3, the mosquitofish was polluted with Cr, Cu, Fe, Mn, Zn, Co and Cd investigated heavy metal in the mentioned sites and months in this study, it will be considered that the levels of Cr, Cu, Fe, Mn, Zn and partially Co, as mentioned above, have been accumulated in the waterbed sediment and bioconcentrated in the *G. affinis* tissues which led to increase heavy metal pollution because, finally they entered in the marine food chains, and biomagnified there after long periods, as confirmed by the Kruskal-Wallis test and Mann–Whitney U-test (Table 4). Previously some studies conducted about heavy metals on *G. affinis*. In a study was proved that Cd body content of the *G. affinis* was increased much higher from water than the food [14,15]. Klerks and Lentz (1998) reported that the tissue metal levels of mosquitofish were highly elevated for lead and (to a lesser extent) for zinc in a contaminated habitat [16]. Franssen (2009) studied the effects of heavy metal mine drainage on population size structure, reproduction, and condition of *G. affinis*. Results showed that the metal contaminated sites had reduced proportions of males and reproductively active females and altered male population size structures [17]. The induction of erythrocyte micronuclei and nuclear abnormalities and Cu and Cd accumulation in whole body of *G. affinis* were studied by Güner *et al*. (2011). When fishes were exposed to Cu and Cd in combination, Cu accumulation was increased compared to alone (0.1 ppm) exposures and erythrocyte nuclear abnormalities were significantly induced [19].

In Iran, Taghavi Jelodar and Hosseinzadeh Colagar (2011) in a study confirmed that the average Cr, Ni, Cd and Pb concentrations were found higher in the female than the male *G. affinis* samples [18]. However statistical analysis doesn’t reveal any significant differences between the average Cr, Ni, Cd and Pb concentrations of females and males. In the present study the levels of Cr, Cu, Fe, Mn, Zn, Co and Cd in the mentioned sites and months were higher than the instrumental detection limits which indicated the *G. affinis* were contaminated with Cr, Cu, Fe, Mn, Zn, Co and Cd among the As, Cd, Co, Cr, Cu, Fe, Hg, Mn, Pb and Zn investigated heavy metal both in females and males mosquitofish simultaneously.

Although some studies confirmed that *G. affinis* can be used as a biomarker or bioindicator such as the blood of the male *G. affinis* has been served as a useful biomarker for assessing previous exposure to estrogenic compounds [11], oxidative stress and locomotor behaviour response of *G. affinis* has been used as biomarkers in the pesticide contaminated aquatic streams [12] and western mosquitofish has been used as a bioindicator of the exposure to organochlorine compounds [13]. This paper confirmed that the *G. affinis* also used as a bioindicator of heavy metal pollution in marine ecosystems such as wetlands. However further studies are needed in this regard.

## Conclusion

In conclusion, *G. affinis* used in many parts of the world to control mosquito larvae [1] for some characteristics such as aggressive nature, high fecundity and major biological impact on many of the ecosystems [13] and can be used as a biomarkers for exposure to estrogenic compounds [11] and pesticide contaminated aquatic streams [12] or bioindicator of the exposure to organochlorine compounds [13], this paper confirmed that the *G. affinis* also used as a bioindicator of heavy metal pollution in marine ecosystems such as wetlands as has been proved due to its widespread occurrence in different regions, easy collection and laboratory cultivation, and its major drawbacks such as highly predaceous habits, they shredding other fish fins, alter zooplankton, insect and crustacean communities, and the relatively low sensitivity to the exposure with contaminants. Although the heavy metals such as cadmium, lead and arsenic can be entered in the Iranian human diets such as rice and tea and cause human health problems [28,29] however *G. affinis* may be fed by the predator such as birds that consumed by human also threatening human health through the food chains. *G. affinis* are well-known and widely used as bioindicator organism in many studies [11-13,19], and if the *G. affinis* populated, it would likely use as a bioremediation of the heavy metal pollution.

## Competing interests

The authors declare that no conflict of interest.

## Authors’ contributions

All authors contributed to the manuscript. All persons listed as authors have read, contributed to preparing the manuscript and attest to the validity and legitimacy of the data and its interpretation, and agree to its submission to “Iranian Journal of Environmental Health Science & Engineering”. All authors read and approved the final manuscript.
